# Post-operative cerebellar mutism syndrome: rehabilitation issues

**DOI:** 10.1007/s00381-019-04229-6

**Published:** 2019-06-20

**Authors:** Philippe F. Paquier, Karin S. Walsh, Kimberley M. Docking, Helen Hartley, Ram Kumar, Coriene E. Catsman-Berrevoets

**Affiliations:** 1 Department of Neuropsychology, University Hospital Erasme, Université Libre de Bruxelles (ULB), Brussels, Belgium; 2 Clinical and Experimental Neurolinguistics, Center for Linguistics (CLIN), Vrije Universiteit Brussel (VUB), Brussels, Belgium; 3 Unit of Translational Neurosciences, School of Medicine and Health Sciences, Universiteit Antwerpen (UA), Antwerp, Belgium; 4 Division of Pediatric Neuropsychology, Children’s National Health System, Departments of Pediatrics and Psychiatry, The George Washington University Medical Center, Washington DC, USA; 5 Discipline of Speech Pathology, University of Sydney, and Sydney Children’s Hospital Network, Sydney, Australia; 6 Department of Physiotherapy, Alder Hey Children’s Hospital, Liverpool, UK; 7 Department of Paediatric Neurology, Alder Hey Children’s Hospital, Liverpool, UK; 8 Department of Pediatric Neurology, Erasmus University Hospital/ Sophia Children’s Hospital, Postbox 2040, 3000 CA Rotterdam, The Netherlands

**Keywords:** Cerebellar mutism syndrome, brain tumor, Rehabilitation, Speech, Language, Ataxia, Behavior, Child

## Abstract

**Introduction:**

Tumors of the cerebellum are the most common brain tumors in children. Modern treatment and aggressive surgery have improved the overall survival. Consequently, growing numbers of survivors are at high risk for developing adverse and long-term neurological deficits including deficits of cognition, behavior, speech, and language. Post-operative cerebellar mutism syndrome (pCMS) is a well-known and frequently occurring complication of cerebellar tumor surgery in children. In the acute stage, children with pCMS may show deterioration of cerebellar motor function as well as pyramidal and cranial neuropathies. Most debilitating is the mutism or the severe reduction of speech and a range of neurobehavioral symptoms that may occur. In the long term, children that recover from pCMS continue to have more motor, behavioral, and cognitive problems than children who did not develop pCMS after cerebellar tumor surgery. The severity of these long-term sequelae seems to be related to the length of the mute phase.

**Aim of this narrative review:**

The impact of pCMS on patients and families cannot be overstated. This contribution aims to discuss the present knowledge on the natural course, recovery, and rehabilitation of children with pCMS. We suggest future priorities in developing rehabilitation programs in order to improve the long-term quality of life and participation of children after cerebellar tumor surgery and after pCMS in particular.

## Introduction

Transient and total cerebellar-induced speechlessness is a complication of cerebellar tumor surgery. Although it has occasionally been reported in adults [11, 35], it is typically considered a pediatric syndrome called the pediatric cerebellar mutism syndrome (pCMS). Its incidence is estimated between 8 and 31% of children undergoing resection of a cerebellar tumor [11]. According to the results of a Delphi procedure and a subsequent consensus meeting of an international group of experts and researchers with a shared interest in pCMS including neurologists, neurosurgeons, oncologists, psychiatrists, neurolinguists, neuropsychologists, speech therapists, and physiatrists, the definition of cerebellar mutism syndrome reads as follows [21]:

Postoperative pediatric CMS is characterized by delayed onset of mutism/ reduced speech and emotional lability after cerebellar or 4th ventricle tumor surgery in children. Additional common features include hypotonia and oropharyngeal dysfunction/ dysphagia. It may frequently be accompanied by the cerebellar motor syndrome, cerebellar cognitive affective syndrome and brainstem dysfunction including long tract signs and cranial neuropathies. The mutism is always transient. But recovery from CMS may be prolonged. Speech and language may not return to normal; and other deficits of cognitive, affective and motor function often persist.

Long-term follow-up studies show that the children who recover from pCMS continue to have motor, behavioral, and cognitive problems, the severity of which seems to be related to the severity of the cerebellar motor syndrome and the length of the mute phase [10, 52, 67]. To date, there have not been any published studies examining and evaluating any specific approach to cognitive remediation or rehabilitation in children suffering from pCMS. In this narrative review, we consider the rehabilitation needs and challenges of treating children with pCMS and associated long-term sequelae, as seen from the perspectives of clinicians in a number of key disciplines who contribute to the care of these children.

## Speech and language disorders in pCMS: clinical presentation and recovery

Mutism is regarded as the hallmark symptom of pCMS, but speechlessness is not always the core symptom of pCMS as patients occasionally present with verbal adynamia or very inhibited verbal output [10, 11]. The latter may be considered to be a part of the wider spectrum of pCMS that includes frontal-like neurobehavioral deficits [10, 11]. Typically after surgery, there is a delayed onset of speech loss after an interval of a few hours up to 11 days of more or less normal speech [10, 11]. Mutism is transient and usually lasts from 1 day to 6 months, but exceptions have been documented [11]. During the mute phase, mutism is limited to speech but other sounds like high-pitched crying and whining or forced laughter are commonly produced [28].

Immediately after the alleviation of the mutism, the presence of dysarthria appears to be the rule. In a study of 27 children with a posterior fossa tumor by Mei and Morgan, the incidence of mutism, dysarthria, and dysphagia post-surgery was reported to affect approximately one in three cases [36]. In a critical review of the literature, De Smet et al. [15] found that 165/167 reliable pCMS cases (98.8%) unquestionably exhibited dysarthria after remission of mutism. Once speech resumes, motor speech deficits often do not display typical ataxic speech symptoms. Van Mourik et al. found slow speech rate to be the most prominent speech feature in a group of children who had undergone cerebellar tumor surgery [63]. Mei and Morgan reported that dysarthria typically involved impairments in articulation, but impacts were also seen across most speech areas including respiration, phonation, and prosody [36]. Furthermore, 63% of children were dysarthric at the time of hospital discharge, and half of these patients had documented comorbid swallowing deficits [36]. The two most distinctive features of ataxic dysarthria—irregular articulatory breakdown and excess and equal stress—are not necessarily the hallmark of post-mutism dysarthria [1, 63]. In a series of 40 children with pCMS, ataxic speech characteristics were only described in five children [10]. In a long-term follow-up study of 24 patients after cerebellar tumor surgery of whom 12 had suffered from pCMS, De Smet et al. observed ataxic speech symptoms in less than 25% [16]. Besides motor speech deficits, a variety of concomitant non-motor language disturbances may manifest after remission of mutism, such as word-finding difficulties (verbal initiation), agrammatism, disrupted language dynamics, comprehension deficits, and reading and writing disorders [17, 18].

Early optimistic claims that dysarthria in pCMS recovers quickly and completely [62] were tempered by more recent and in-depth follow-up studies. Children are often left with persistent dysarthria, language impairment, and dysphagia, among other neurological deficits [15, 18, 21, 36]. In their long-term follow-up study, De Smet et al. found that all children with pCMS exhibited dysarthria in the immediate post-mutism phase, and 91.7% of them had persistent motor speech deficits up to 12 years after surgery [16]. They confirmed the findings of Huber et al. [25] of persistent motor speech defects in a long-term follow-up of more than 5 years, and endorsed the view that pCMS is a prognostic factor for long-term dysarthria in children treated for cerebellar tumors [16, 25].

A number of key prognostic factors have been identified for the development of pCMS. Di Rocco and colleagues [18] showed that specific impairment of speech and language functions can be present even at diagnosis of a posterior fossa tumor. Specifically, pre-operative language impairment was noted to be a strong risk factor for pCMS. Pre-operative language impairment was characterized by reduced spontaneous language and decreased mean length of utterance. In addition, specific disorders were noted in tasks assessing word-finding abilities and the ability to generate words according to a given phonological rule. The authors noted that pre-operative language impairment appears to signal the appearance of cerebellar mutism [18]. Invasion of the brainstem, rather than compression, was also noted to be a prognostic factor in relation to the onset of pCMS [18]. Additionally, while hydrocephalus was not identified as significantly different across groups, the incidence of persistent hydrocephalus was strongly correlated with language impairment. These findings strongly indicate the need for careful assessment of speech and language both prior to and following surgery. Advanced knowledge of these factors would assist in optimizing the multi-disciplinary management of pCMS and assist in conveying information related to risk to parents and families. Referral to a speech-language pathologist for a comprehensive and systematic assessment of speech and language in children with cerebellar tumor is vital in order to facilitate the early detection and management of pCMS, with a view to reduce negative long-term consequences [36].

## Rehabilitation of speech and language problems during and after the mute phase

There is no established treatment for the speech and language disorder of pCMS to date. Attempts have been made to reverse mutism in its acute stage by medication intervention. Corticosteroids, fluoxetine, thyrotropin-releasing hormone, bromocriptine, midazolam, and zolpidem have all been used with inconsistent results [11]. At present, the complex array of post-operative challenges for children with pCMS requires an integrated approach to rehabilitation. Early intervention is required with a long-term plan for the management and surveillance of speech and language, as children remain at risk throughout development [15, 64, 65]. As a group, children receiving surgery and other treatments for brain tumors often experience poor long-term quality of life outcomes due to disorders of speech and language, which include academic failure and loss of friendships that lead to devastating negative impacts upon healthy development and socialization [2, 19, 33, 43, 51]. In addition to the effects on existing speech and language functions, skills that are yet to develop are also considered vulnerable, with children failing to acquire or develop skills at the expected rate over time over development [65]. Early intervention in children treated for brain tumors has been reported to effectively minimize speech and language deficits [59].

Routine clinical management should be central to the rehabilitation of children with pCMS, with a systematic integrated approach to the management, assessment and treatment of speech and language disorders, and follow-up and early intervention. It is therefore recommended that rehabilitation of speech and language in children with pCMS involves multi-phase implementation of evidence-based guidelines and recommendations that identify and highlight risk factors, specific deficits, and knowledge about progression over time [9, 13, 38]. In summary, it is recommended that integrated, holistic rehabilitation of pCMS involves the following stages:

1. Establish a pre-operative baseline of speech and language function, including identification of existing risk factors/predictors.
2. Identify the nature of speech and language disorders occurring in the post-operative period, including presenting signs and symptoms, and speech and language characteristics.
3. Determine the nature of speech and language rehabilitation required, such as timing of intervention, speech and language rehabilitation, and referral.
4. Follow-up during the recovery process, defining intermediate assessment intervals, including recommendations for surveillance across developmental time points, long-term recovery, and communication with the patient and family.
5. Identify risks of late effects on speech and language, where children tend to grow into their deficits, and the impact on quality of life.

## Behavioral and cognitive disturbances during pCMS and course of recovery

In the acute phase of pCMS, children initially are extremely irritable and suffer from mood lability. They usually are either severely apathetic or indifferent to their surroundings and can show inconsolable crying and whining behavior. More rarely, these children exhibit disinhibition with forced laughter. Compulsive pre-sleep behavior, cortical blindness, and impaired voluntary eye opening (eyelid apraxia) may also be present. The most severe behavioral disturbances almost always slowly diminish before the end of the mute phase and the return of speech [10, 11].

There have been a number of studies reporting on the longer-term neurocognitive outcomes of children with brain tumors with pCMS. The literature is clear that children diagnosed and treated for tumors involving the cerebellum demonstrate a range of neurocognitive and neuroemotional deficits in the years following treatment [52, 67]. The exact presentation is associated with a combination of factors including the location of the tumor in the cerebellum, treatment approaches (e.g., radiation), and other factors such as hydrocephalus [37, 41, 48, 49]. The functions predominantly disrupted in this population of children include functions which are thought to be processed in the frontal part of the cerebral hemispheres such as processing speed/cognitive efficiency, executive functions, attention, visual-spatial and perceptual functions, language, affect, and behavior [37, 41, 48, 49]. The common long-term deficits seen in children with cerebellar tumors have been noted to have consistencies with the cerebellar cognitive affective syndrome (CCAS) originally described in adults with cerebellar insults [31, 56, 61].

Beyond the impact on neurocognitive, affective, and behavioral functioning in children with cerebellar tumors more globally, children with pCMS have been described to demonstrate even greater morbidity in these domains as early as 1 year after diagnosis [31, 39, 42, 66]. Significant differences between children with cerebellar tumor with and without pCMS have been documented with the children with pCMS having significantly lower performance in global intellect [31, 39, 42, 66], processing speed, attention, executive functions, language, memory, academic achievement, and emotional regulation [1, 31, 42]. Children with more severe pCMS, as operationalized by Robertson et al., demonstrate more lasting neurocognitive, affective, and behavioral morbidity with less recovery over time [52].

## Treatment and rehabilitation of behavioral and cognitive problems during and after pCMS

Children who present with pCMS are at substantial risk for greater long-term cognitive and psychosocial morbidity, making them an important group for which interventions and rehabilitation beyond those focusing exclusively on motor and speech functions should be prioritized in the scientific community. The primary areas of rehabilitation therapy that these children receive target the motoric and speech/language deficits that are observed in the acute period around pCMS via physical, occupational, and speech/language therapies. It may be that cognitive interventions are not implemented in the acute phases of pCMS because the cognitive issues (beyond the obvious disruption in ability to communicate) do not become evident until later. However, there may in fact be opportunities to alter the eventual emergence of these sequelae if we intervene earlier. There have been three primary areas of research targeting neurocognitive impairment in pediatric cancer patients (e.g., brain tumor and leukemia), and while these studies have not specifically targeted children with pCMS, a review of this literature may be prudent to the future development of cognitive rehabilitation research for these children in particular.

### Pharmacological interventions

Stimulant medications have been the most researched pharmacologic approach to addressing cognitive deficits in pediatric cancer survivors to date. Two randomized clinical trials of stimulant medication in pediatric cancer survivors (predominantly brain tumor) have reported positive results. Improvements in performance-based attention as well as improved focus based on parent and/or teacher reports were found [40, 61]. In addition, improvements in academic competence and social functions were also reported [40].

Neurotransmitter modulators targeting memory processes have also been evaluated. Castellino and colleagues published a single-arm, open-label trial of donepezil in a small sample of pediatric brain tumor survivors [8]. Following 24 weeks of treatment, significant improvements were noted in visual memory and aspects of executive function. Despite limitations in this study (e.g., small number of subjects, lack of comparison group), there may be promise in this line of research. There is a randomized clinical trial of donepezil in adult brain tumor survivors with some evidence of potential benefit in memory function, particularly for those that were more impaired at baseline [46].

### Cognitive rehabilitation

Research on cognitive remediation programs fall into two distinct categories: (1) face-to-face therapeutic sessions targeting specific functions such as executive function, attention, memory, and academic achievement, and (2) computer-based intervention programs that are implemented within the home setting. The most comprehensive cognitive remediation research program to date was a phase 3 clinical trial of a multi-dimensional program of interventions targeting (a) hierarchically graded massed practice, (b) strategy acquisition, and (c) cognitive-behavioral therapy. The results indicated significant improvements in academic achievement, attention, and improved implementation of metacognitive skills [7]. This type of intensive intervention program, while promising with regard to potential benefit, is marred by problems with attrition as families find it difficult to be able to make the time commitments necessary for this type of intervention.

A number or recent studies have examined the feasibility, acceptability, and efficacy of computer-based, in-home cognitive intervention programs in pediatric cancer survivors. Two published studies specifically using CogMed, a computerized intervention program targeting working memory, have shown good feasibility, acceptance, and compliance (85–88%) in pediatric cancer populations including brain tumor patients [14, 22]. In addition, improvements in visual working memory and parent-reported learning difficulties were reported, with the greatest improvement observed in children with higher intellectual functions prior to treatment [22]. There is some indication that this intervention may also result in increased activation on fMRI during a sustained attention task in pediatric cancer survivors following intervention in brain regions shown to be active in a healthy comparison group [69].

### Physical exercise

Two studies have been published to date and others are in progress evaluating the effect of exercise programs in pediatric brain tumor survivors [5, 50, 60]. In a controlled crossover exercise program study in long-term survivors of pediatric brain tumors treated with radiation, there is some evidence of structural brain changes including increased white matter and hippocampal volume and increased cortical thickness in multiple brain regions [50, 60]. Additionally, improvements in reaction time were demonstrated [50].

Each of these primary areas of neurorehabilitation research have yet to be applied specifically to the population of children with pCMS. However, these intervention approaches may hold promise for addressing the specific neurocognitive sequelae in children with pCMS specifically given that they have been found to suffer more intense long-term consequences than pediatric brain tumor survivors without pCMS.

## Motor disturbances in the context of pCMS

Children who present with pCMS can demonstrate a range of motor disturbances including hypotonia, hemi- or tetraplegia, ataxia, and cranial nerve palsies. The most common motor difficulties seen are known as the cerebellar motor syndrome which incorporates ataxia and postural/gait deficits [48]. Ataxia and balance problems can affect activities of daily life, return to school, and participation with peers, with truncal ataxia thought to have the most significant impact on functional ability [34, 45].

Ataxia is often present in children pre-operatively (reported as evident in 60% of children in a meta-analysis by Wilne et al. [68]). Ataxia is then thought to increase post-operatively with pCMS highlighted as a risk factor for both more severe [23] and long-term ataxias [29]. Specific tumor location is also noted to be a risk factor for long-term ataxia with involvement of the deep cerebellar nuclei, a predictor for problems over a year after surgery [29]. Overall, there is a lack of understanding regarding the recovery potential for children with motor problems in conjunction with pCMS although recent studies suggest that children who were diagnosed with pCMS are likely to have more often long-term therapy needs [23, 27].

## Rehabilitation of cerebellar motor problems during and after pCMS

Therapy management remains the mainstay of treatment for motor problems for children with pCMS. Physical rehabilitation is typically delivered as part of a multidisciplinary approach to optimize care, while providing a tailored program with individualized goal setting involving the child’s family [20]. The presence of pCMS often provides challenges of integrating physical rehabilitation in the context of emotional, cognitive, and communication problems, and therefore, therapy sessions are flexible and responsive to the child’s presentation and coordination with other therapists is crucial.

Aside from emerging evidence for its benefit on cognitive outcomes [5, 60], there is a particular lack of literature on physical rehabilitation for children with pCMS to improve motor outcomes. However, there is evidence of functional improvement in children with brain tumors in general following acute inpatient multidisciplinary rehabilitation [44]. Regarding physical rehabilitation for children specifically with ataxia in conjunction with pCMS, there is again a lack of evidence on the most beneficial type and intensity of physical rehabilitation. However, there is a suggestion of improvement with conventional physical therapy (for example, strengthening exercises, balance training, practice of functional tasks) for children with ataxia in the wider context of traumatic and acquired brain injury [26, 54].

Sabel et al. report that home-based video gaming can improve balance in children after brain tumor treatment [53]. Ada et al. in a single-case study of a girl with upper limb ataxia following resection of a posterior fossa tumor reported short-term but not significant improvements in upper limb timing following a computer tracking training program [3]. There is also a suggestion of benefit from treadmill training for children with balance problems as their primary impairment in children with acquired brain injury and cerebral palsy which may also be useful to consider as an adjunct to therapy treatment [12, 47]. Ongoing assessment of children’s physical ability and function with pCMS is also important to track progress and assist with development of their therapy program. Outcome measures that may be used include the pediatric SARA (Scale for the Assessment and Rating of Ataxia) [30, 58] or BARS (Brief Ataxia Rating Scale) [57], which enable a quantification of severity of ataxia. These may be used alongside more functional and participation/quality of life measures, for example the Bruininks-Oseretsky test of Motor Proficiency 2 [6] or the Pediatric Evaluation of Disability Inventory [24].

## Conclusions and thoughts on further research

Post-operative pCMS is a prevalent and deleterious complication of cerebellar tumor surgery that affects children far more often than adults [11, 35]. It is transient in nature and typically turns into dysarthria once speech resumes after the mute phase. Transient cerebellar mutism is a symptom embedded in a constellation of associated devastating neurolinguistic, neurocognitive, and neurobehavioral and motor disturbances, many of which persist and even worsen over time [10, 11, 52, 67]. It is of utmost importance to prepare parents and caregivers of children that have surgery for a posterior fossa tumor for the risk that their child will develop pCMS and inform them on the variety and severity of symptoms that may occur. Identification of existing risk factors/predictors for pCMS is essential and may include a pre-operative comprehensive baseline assessment of speech and language [18] and the use of a pre-operative MRI-based scoring scale [32].

The profound, persistent, and widespread impact of pCMS highlights the importance of ongoing rehabilitation and effective and early intervention [42, 52, 67]. The complexities of pCMS stress the importance of multidisciplinary management [64]. In the case of pCMS, restoration of motor functions and communication relies heavily upon physiotherapy and occupational, speech, and language therapy [64]. Rehabilitation in the acute phase of pCMS is often severely hampered by the behavioral deficits that prevent the child from fully participating in therapy at a level necessary for effective rehabilitation. An integrated approach to rehabilitation that also includes in the team physiatrists, neuropsychologists, child psychiatrists, and social workers—in close cooperation with the family—will serve to improve the quality of life for children with pCMS, as well as their families and wider communities. Walker and colleagues described families and the school community as “powerful agents of change,” where family-delivered rehabilitation is considered much more effective than clinician-directed management [64].

In patients with pCMS, presenting signs and symptoms and later speech and language characteristics should be analyzed in order to determine the nature of speech and language rehabilitation required, such as timing of intervention, speech and language rehabilitation, and referral. Focused neuropsychological assessments of specific areas of functioning can be beneficial in the more acute phases (e.g., within the first year after diagnosis). The selection of assessment tools used in these early focused assessments requires critical evaluation of tools that are adequately sensitive and consider issues of practice effects and psychometrics. The primary targets of these early focused assessments should include the evaluation of communication, attention, executive functions, visuomotor skills, and psychological functioning. More comprehensive neuropsychological evaluation becomes of greater benefit in the long-term follow-up of patients with pCMS (beginning at 12 months after treatment has been completed). In addition to the cognitive domains evaluated in the focused assessments, comprehensive long-term monitoring should also include the evaluation of memory functions, reasoning skills, processing speed, and adaptive functions.

Medication has not been consistently utilized to alleviate symptoms in acute pCMS or in the more chronic stages in which pCMS symptoms are consistent with CCAS. Future research is necessary to determine how effective this approach may be and for what symptoms. Potential targets for future research in this area may build on the preliminary data that has been presented on the use of such medications.

During recovery, assessment intervals should be defined, including recommendations for surveillance across developmental time points, long-term recovery, and communication with the patient and family. In addition, the risks of very late effects of pCMS in terms of growing into deficit during further development should be identified and timely addressed. Cognitive rehabilitation after pCMS should be tailored to the individual needs of the child, and evaluations are important in developing such individualized rehabilitation or interventions. Where children are left with significant residual impairment, the incorporation of compensatory technology to aid communication is recommended [64]. Interventions, particularly those focused on the cognitive consequences of pCMS, should consider the use of telemedicine or alternative intervention delivery (e.g., home-based computer intervention programs) to maximize access and feasibility to these interventions. In addition, the emerging literature on the positive impact of physical exercise programs in pediatric brain tumor survivors also supports this as a target for future research and clinical implementation [5, 50, 60].

Alternative procedures such as transcranial magnetic stimulation (TMS) and transcranial direct current stimulation—especially in combination with cognitive interventions—may be promising techniques to stimulate neurorecovery and plasticity in adults [55]. TMS has been found to be safe in children and adolescents with central nervous system disorders [4]. Further exploration of the effect of TMS alone or combined with a cognitive intervention in children with pCMS also may lead to new opportunities.

Given the paucity of controlled data on the therapeutic options, there is a need for systematic prospective trials evaluating the effects of different neurosurgical approaches, pharmacological interventions, neurorehabilitation, and other alternative strategies for preventing and addressing the sequelae in children with pCMS. Achieving a balance between optimal survival and minimal long-term sequelae will ultimately result in an improved quality of life and functional outcomes of children after brain tumor treatment and more specifically after pCMS.
